# Efficacy of echolaser smart interface-guided laser ablation in volume reduction of symptomatic benign thyroid nodules

**DOI:** 10.3389/fendo.2024.1402522

**Published:** 2024-10-09

**Authors:** Iftikhar Malik, Janeil Mitchell, Johnson Thomas

**Affiliations:** ^1^ Department of Endocrinology, Fox Valley Surgical Specialists, Appleton, WI, United States; ^2^ Division of Endocrine Surgery, Department of General Surgery, Fox Valley Surgical Specialists, Appleton, WI, United States; ^3^ Department of Endocrinology, Mercy Hospital, Springfield, MO, United States

**Keywords:** laser ablation, thyroid nodule, echolaser, benign nodule, ablation

## Abstract

**Background:**

The management of benign symptomatic thyroid nodules until recent years has been limited to surgery, radioactive iodine treatment, or surveillance which is associated with the burden of morbidity of complications or symptom non-relief as well as cost. Laser ablation has emerged as a minimally invasive alternative, this uses laser energy to thermally ablate nodule tissue, leading to volume reduction and symptom relief. Long-term treatment response data is growing but remains limited in the United States. Our study aims to quantify the effectiveness of laser ablation in reducing the volume of thyroid nodules over a 12 to 18-month period.

**Materials and methods:**

Retrospective review of data was conducted for 63 adults with cytologically benign, solid symptomatic thyroid nodules ranging from 1.333 cm^3^ to 103.794 cm^3^ in volume. Ultrasound-guided laser thermal ablation was performed on all nodules using EchoLaser X4 Smart Interface device with 1064 nm diode laser to deliver total ablation energy (joules), calculated per device guidelines. Serial sonographic volume measurements were conducted 1 month, 3 -6 months, 6 - 12 months, and 12 to 18 months post-ablation intervals.

**Results:**

Study cohort was comprised of 63 thyroid nodules. reduction in nodule volume increased progressively over time, with median reductions of 46.05% [STD 21.8] at 1 month, 60.33% [STD 20.1] at 3-6 months, 68.69% [STD 18.8] at 6-12 months, and 64.04% [STD 19.27] at 12-18 months. A total of 62, 56, 42, and 17 nodules had available data for analysis at these respective intervals.

**Conclusion:**

This study demonstrated a marked progressive reduction of thyroid nodule volume following ablation. The treatment appears to be consistently effective in reducing symptoms across a wide range of nodule sizes, although the degree of volume reduction varies. The results of our study underscore the potential of laser ablation as a viable treatment option for thyroid nodules, with a sustained reduction in nodule volume observed over an extended post-procedure period.

## Introduction

Laser thyroid nodule ablation is a minimally invasive percutaneous technique utilized in the treatment of symptomatic, functional, and non-functional benign thyroid nodules (1). The incidence of thyroid nodules among the general population is on the rise, with up to 1 in 10 benign nodules observed to be symptomatic over an individual’s lifetime, the number of patients potentially needing therapeutic intervention is also rising (2, 3). Historically, surgery has been the sole treatment option for solid nodules causing local discomfort or aerodigestive compression, but over the past two decades, percutaneous image-guided thermal ablation modalities including radiofrequency ablation, cryoablation, and microwave ablation, high-intensity focused ultrasound have emerged in as a potential treatment alternatives.

Laser thyroid ablation involves the application of thermal energy via a thin optical fiber inserted percutaneously into a target thyroid nodule creating a predictable zone of coagulative necrosis. Laser ablation has been shown to significantly reduce the volume of thyroid nodules leading to reduction of compressive symptoms and improved cosmetic outcomes for patients (4–7).

Clinical data continues to grow, illuminating procedural challenges and areas of research. One limitation to treatment efficacy in thermal ablation is possible regrowth of nodule tissue at ablated margins and need for retreatment for insufficient volume reduction and symptom control (8–10). Contributing factors are unclear, however commercial laser devices have developed image guidance packages to aid in volumetric ablation planning.

The primary objective of this study was to assess the effectiveness of percutaneous laser ablation under an ultrasound guidance platform with spatial overlay for treatment planning,[Echolaser Smart Interface] in volume reduction of symptomatic, benign thyroid nodules.

## Materials and methods

### Study design and data collection

This is a single center retrospective study. A retrospective electronic medical chart review was conducted at an outpatient endocrinology clinic, Fox Valley Surgical Specialists (FVSS) to identify all patients who were treated with laser ablation for thyroid nodules from January 2020 to December 2022.

Inclusion criteria included all adult patients undergoing laser ablation at FVSS. Patients lost to follow up after laser ablation without at least one follow up were excluded from the study.

Data collected from the charts included patient demographics (age and sex), results of fine needle aspiration biopsies, laboratory values including TSH, Free T4, and Free T3, and measurements from thyroid and neck ultrasound images. Baseline assessments, as documented in the chart were also collected and entered into a spreadsheet. This included patient history, physical exam, and thyroid nodule symptom assessment. Measurements of the nodule from the same day pre-ablation and post-ablation using ultrasound, TSH, Free T4, Free T3 values, as well as clinical notes on pre- and post-ablation symptoms and procedural complications were also collected. Thyroid nodule symptoms were assessed using questions with binary answers.

The study was approved by the Pearl IRB (2023-0298-DFT).

Changes in thyroid nodule volume in cm3 were measured using ultrasound at 1 month, 3 to 6 months, 6 to 12 months and 12 to 18 months after laser ablation. All descriptive and discrete data points were collected and entered into a password-projected computerized spreadsheet.

### Laser ablation

Laser ablation procedures were completed in an outpatient endocrinology clinic under sterile conditions. The ablation site was thoroughly cleansed with antiseptic solution and sterile drapes were applied to create a barrier around the surgical field. Ultrasound probes were covered with sterile probe covers. The healthcare providers donned sterile gowns and gloves before entering the sterile field. Only sterile instruments and materials, including needles, were introduced into the field. Standard laser precautions were followed.

The Elesta ECHOLASER X4 laser system, developed by ElestaSpA based in Calenzano, Italy, was employed, augmented by its accompanying guidance software[Echolaser Smart Interface] (1). This system was approved for clinical use from the United States Food and Drug Administration (FDA) under the 510(k) Premarket Notification pathway on September 4, 2018.The ECHOLASER X4 was utilized with a single diode laser source operating at a wavelength of 1064 nm and can deliver a maximum power output of 7 watts.

Total Laser energy for the nodule was calculated using device guidelines. Average energy delivered per nodule was 3461 Joules.

During thyroid nodule ablation, ultrasound guidance was utilized continuously during the ablation planning phase, needle insertion, and continuous monitoring of the ablation process. The introduction of the fiber into the thyroid nodule was facilitated using a 21-gauge needle introducer. The Echolaser Smart Interface (ESI) was employed to aid in the accurate placement and positioning of the needles into the target lesion. Laser energy transmission to the target site was achieved through the use of bare flat-tipped quartz optical fibers with a diameter of 300 μm, sourced from Oberon GmbH, Wildau, Germany

In all patients, local anesthesia using 1% Lidocaine without epinephrine was used. Hydrodissection technique was used not only for anesthesia but also to establish a protective buffer zone between the thyroid and vital structures, including the carotid arteries, jugular veins, and trachea. Our ablation approach primarily involved a trans-isthmic technique. As the targeted area underwent ablation, the fibers were repositioned to a fresh area. Nodule ablation was initiated from the inferior aspect and systematically progressed in an upward direction by the pullback technique (1).

Upon the conclusion of laser ablation, the removal of the fibers was carried out, accompanied by the extraction of the needle(s), and hemostasis was accomplished through the application of pressure at the respective site. Subsequent to the needle extraction, an evaluation of external bleeding was conducted through visual inspection. Furthermore, an assessment of internal bleeding and the potential for nodule rupture was performed by the investigator using neck ultrasound with Doppler flow.

Pain and complication evaluations were conducted immediately following Laser ablation and documented in the medical record. Subsequent follow-up assessments were scheduled at 1, 3, 6, and 12-month intervals following the conclusion of the study procedure. These follow-up visits encompassed comprehensive evaluations, including medical history reviews, physical examinations, and complete ultrasound of the neck with Doppler assessment. The continuous monitoring of adverse events was an integral part of the study protocol. Two investigators (IM or JM) conducted all physical examinations, data collection, ultrasound examinations with Doppler imaging, and administered the laser ablation procedure for all enrolled subjects.

### Data collection and analysis

Neck ultrasound examinations, incorporating Doppler imaging, were conducted utilizing the GE Logiq P9 ultrasound system and ML6-16 probe. The volume of thyroid nodules, measured in milliliters, was determined using the formula: Nodule Volume = Length (cm) × Width (cm) × Depth (cm) × 0.525 (11). To assess the change in nodule size, the percentage reduction in nodule volume was calculated via the equation: (1 - Final Volume/Initial Volume) x100 (12). Additionally, thyroid function tests and symptomatology, both pre- and post-procedure were recorded and analyzed.

Data analysis and generation of descriptive statistics was completed using Python statistical libraries (13). Mean, median volume changes during each interval, standard deviation, interquartile range, change in volume for each interquartile range over each time interval specified in the study were calculated. Efficacy of laser ablation was assessed by the change in volume over time after laser ablation.

## Results

In this study, we evaluated the efficacy of laser ablation in reducing the volume of cytologically-benign solid thyroid nodules. The analysis encompassed data from various time intervals post-procedure: 1 month, 3-6 months, 6-12 months, and 12-18 months. A total of 62, 56, 42, and 17 nodules had available data for analysis of these respective intervals. Sixty-three patients were included in the study, consisting of 58 women and 5 men. Benign functioning and non functioning nodules were included in the study. One patient was lost to follow-up.

There were 58 females and 5 males in the study cohort with ages ranging from 26 to 85 years. Median age was 61. Pre-procedure nodule volume ranged from 1.333 cm^3^ to 103.794 cm^3^. Median volume was 9.595 cm^3^.

**Figure 1:**
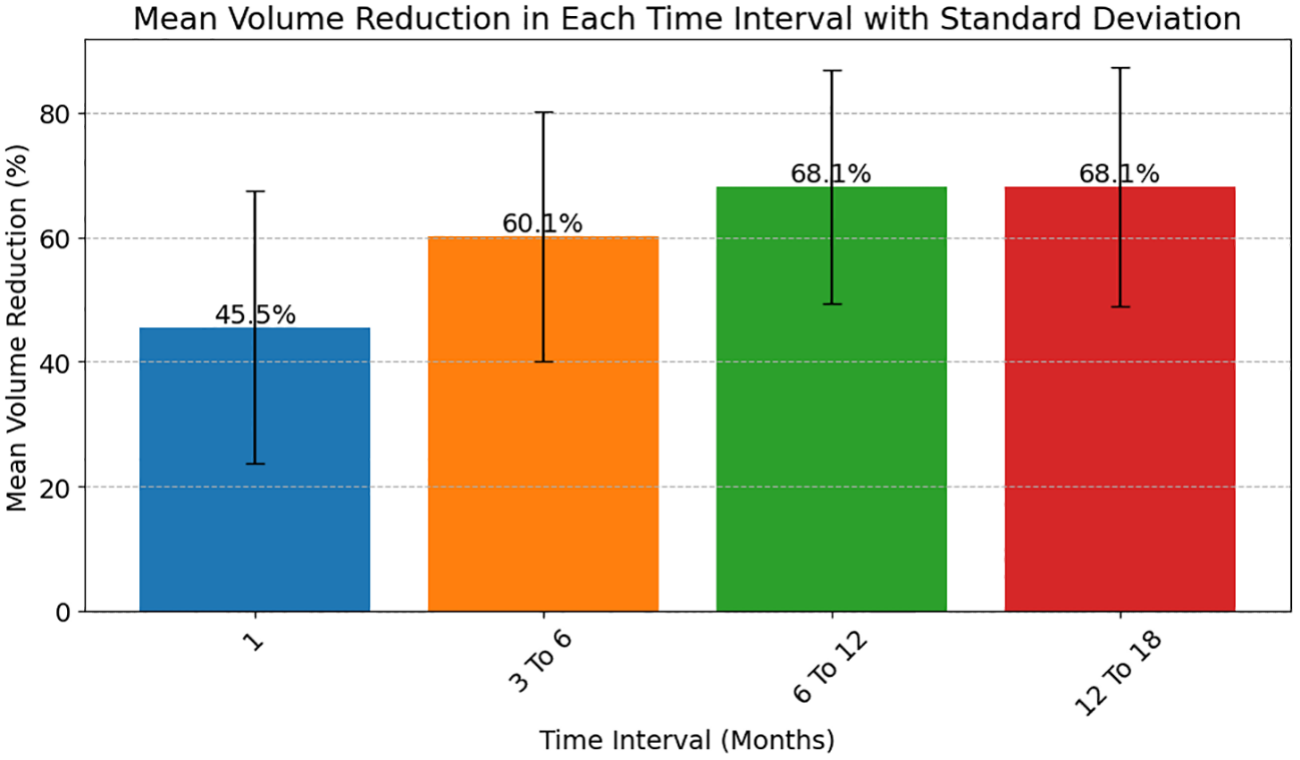
Post-ablation mean volume reduction in thyroid nodule volume at different time intervals.

The median reduction in thyroid nodule volume was 46% [STD 21.8] at 1 month and observed to increase progressively over time. **Figure 1** illustrates the mean percentage reduction in thyroid nodule volume over sequential post-ablation intervals following laser therapy.

Four distinct time frames are reported in **Figure 1**: 1 month (blue box), 3-6 months (orange box), 6-12 months (green box), and 12-18 months (red box) post-treatment. The bar corresponding to the 1-month interval exhibits a 45.5.% reduction in nodule volume, indicating initial response to the ablation procedure. Subsequent time intervals show progressive volume reduction, with the 3-6 months interval displaying a 60.1% decrease, the 6-12 months interval a 68.1% decrease, and the 12-18 months interval demonstrating a 68.1% decrease.

**Figure 2:**
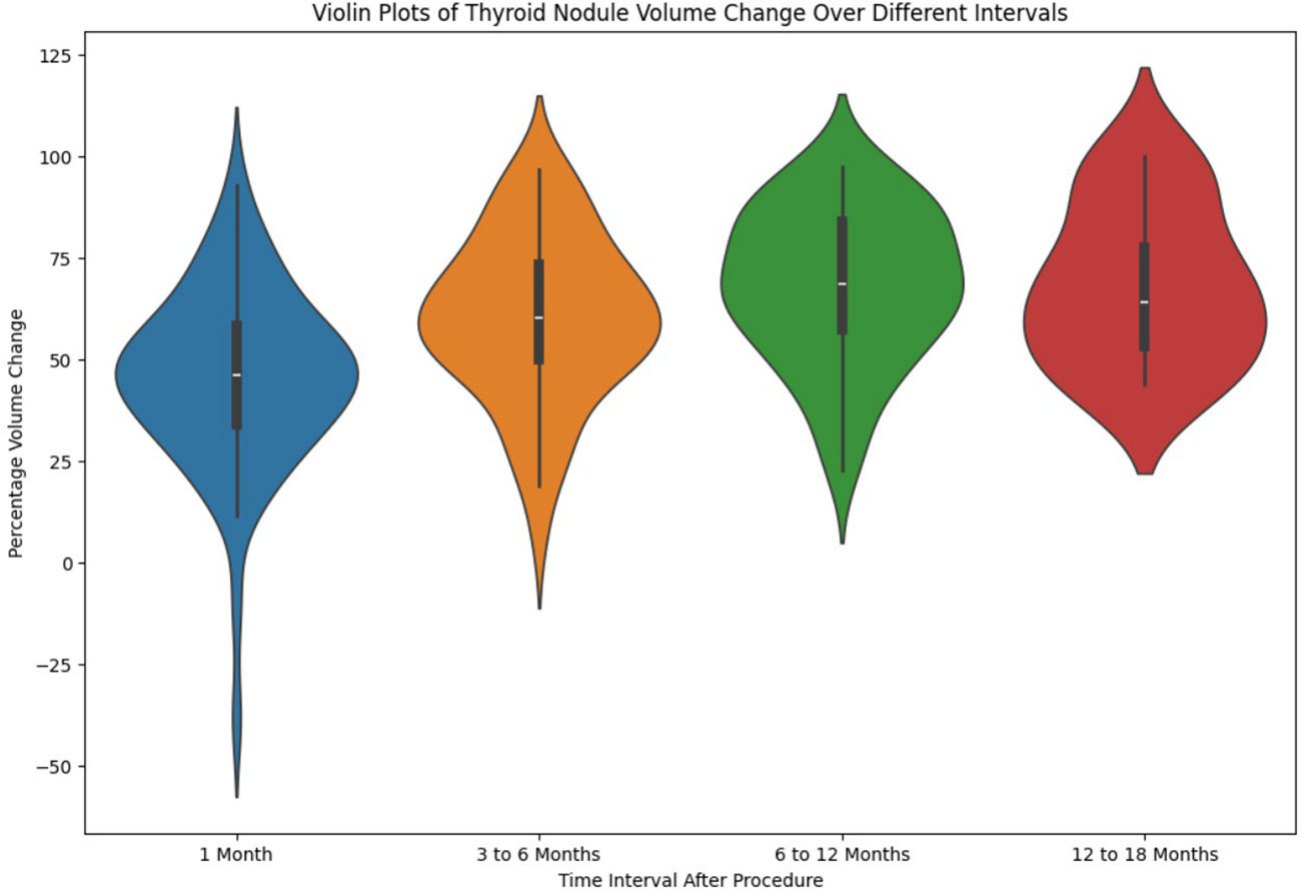
Distribution of thyroid volume reduction over different time intervals.

The distribution of percentage reduction in thyroid nodule volume across the four post-ablation time intervals: 1 month, 3-6 months, 6-12 months, and 12-18 months is presented as a violin plot in **Figure 2**. Each ‘violin’ encompasses a kernel density estimation illustrating the spread and probability density of the data at different percentage reductions. The central boxplot within each violin indicates the interquartile range (IQR) with a line marking the median percentage reduction.

The first violin(blue violin) for the 1-month interval shows a median reduction close to the 50% mark, with the majority of the data points spread around the median, indicating a consistent response among patients. The second violin (orange violin) for the 3-6 months interval displays a slightly higher median reduction, suggesting a variation in the response among patients as time progresses. The third violin (green violin) for the 6-12 months interval demonstrates a median that is higher still, with a broader distribution. The fourth violin (red violin) for the 12-18 months interval shows similar median reduction as the 6-12 month plot.

### Volume reduction: subgroup analysis based on initial nodule volume

**Figure 3:**
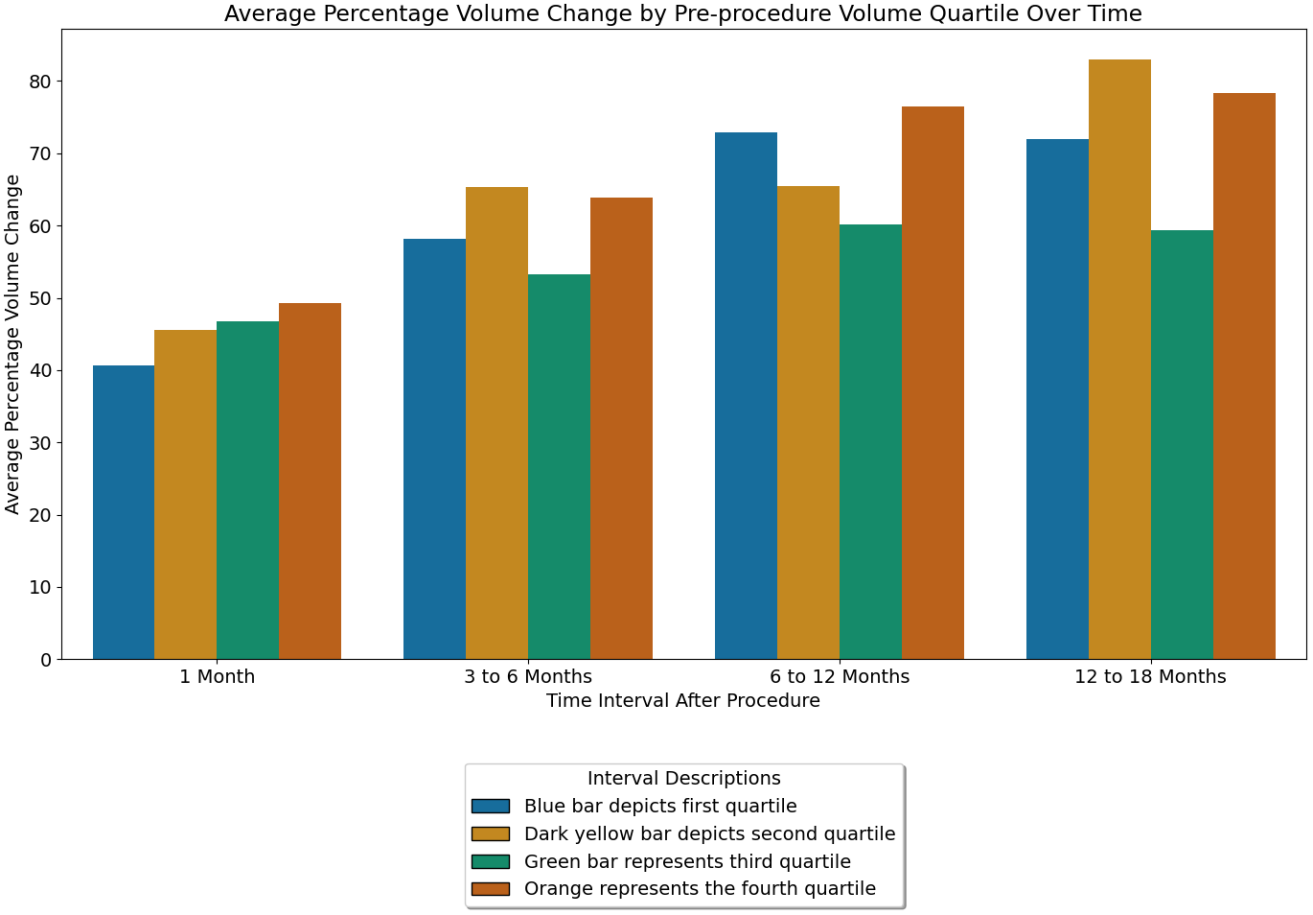
Mean percent volume change by pre-procedure volume quartile over time.

**Figure 3**, bar clusters revealed a few notable trends. At the 1-month interval, all quartiles demonstrate significant volume reduction. There was progressive volume reduction with increasing quartile at this stage. The 3-6 and 6-12 months interval shows a continued increase in volume reduction. But there was no statistically significant difference between the quartiles.

No immediate post procedure complications bleeding, difficulty swallowing were noted. No hospitalization after LA was reported. There was no clinically relevant change in thyroid function after laser ablation in patients with normal thyroid function. No patient had recurrent laryngeal, tracheal or esophageal damage. No subcutaneous burns or vascular damage occurred during or after the procedure. For patients not lost to follow-up (62 patients) and who had final symptom assessment, the majority of patients reported symptoms either resolved (n = 46, 85%) or improved (n=9, 16.6%) after LA. Of the 63 patients, 5 patients (7.9%) underwent second LA due to inadequate resolution of compressive symptoms. Of these 5 patients, the average volume reduction was 67.56% at 12 months.

## Discussion

In this study, the Elesta Echolaser was employed to ablate benign symptomatic nodules, producing progressive reduction of nodule volume from initial ablation up to 12 months, and sustained mean reduction at 18 months. Initial volume reduction, within the first month, was most pronounced for the largest nodule. We observed an average volume reduction of approximately 12 cubic centimeters at 12 months following laser ablation of thyroid nodules. This aligns with findings from Rahal Júnior et al., where a reduction of about 60% in thyroid nodule volume was noted after a year (14). Average pre-procedure volume was 1.67cm3. Our subgroup analysis, which categorized nodules based on their initial volume, revealed differential responses to treatment. In the first month after treatment, volume reduction was proportional to the preprocedural volume. But later on in the study this trend disappeared. It is important to note that the correlation between pre-procedure nodule volume and percent reduction was generally weak, indicating that while size may influence the response initially, it is not the sole determinant of treatment success. Other potential factors that could affect the volume reduction includes, amount of energy applied and composition of the nodule (15).

Not all thyroid nodules are amenable to currently available minimally invasive procedures. Cystic or predominantly cystic nodules are best treated with aspiration or ethanol ablation (16). Nodules extending into the mediastinum may not be fully accessible percutaneously, and risks pneumothorax. Some researchers have reported regression of volume reduction long-term, thought to possibly represent inadequate margin ablation, leading to the possibility of recurrence. Since laser ablation relies on heat to destroy tissue, it can be difficult to definitively assess the success of the procedure during the treatment itself. Some centers in Europe and Asia use contrast enhanced ultrasound after ablation to identify the area of ablation (17). But this is not approved for use in the United States. We employed echolaser smart interface to aid in uniform spatial ablation using pullback technique along paths within a grid overlaying the thyroid nodule. The average percent reductions observed at 1 month (45.54%), 3-6 months (60.1%), 6-12 months (68%), and 12-18 months (68.1%) in our study are comparable to those reported in previous research (9, 18–21). Our study had only 63 participants and not all patients had 18 month follow up. It is unknown if the treatment effect will continue long-term, but results underscore the potential of laser ablation as an effective treatment for reducing the size of thyroid nodules. In recent years, a growing body of literature has supported LAs efficacy and safety. Studies have consistently reported significant reductions in nodule volume, improvement in local symptoms, and a low rate of complications (22). Our findings align with this emerging consensus, demonstrating a progressive decrease in nodule volume over 18 month period following laser ablation without significant complications (23).

In 2020 European Thyroid Association reviewed available data on thyroid thermal ablation techniques and published clinical guidelines (24). According to this guideline, image guided thermal ablation can be offered to adult patients who have pressure or cosmetic symptoms. This guideline also recommended dedicated training for the operator and discussion of potential side effects with the patients. A recent American Thyroid Association multidisciplinary consensus statement acknowledges the increasing adoption of thermal ablation techniques and calls for safe adoption and implementation in the United States (25). A pilot study in the United States demonstrated safe and effective Laser ablation in an outpatient setting (26).

As the prevalence of thyroid nodules increases, particularly in aging populations, treatments that offer reduced recovery times, lower complication rates, and preservation of thyroid function become increasingly valuable. Laser ablation fulfills these criteria, providing a viable option for patients who may not be ideal candidates for surgery or who prefer a less invasive approach to avoid surgical risks including laryngeal nerve injury (27). Minimally invasive procedures also have the potential to reduce healthcare spending. Studies comparing cost effectiveness of minimally invasive procedures to open thyroidectomy has shown potential significant cost savings (28, 29).

Our study contributes to the growing body of evidence supporting the use of laser ablation for the treatment of thyroid nodules. The observed reductions in nodule volume over time, coupled with the treatment’s minimal invasiveness, position laser ablation as a promising alternative to surgery for suitable candidates. Future research should focus on multi-year long-term outcomes, the identification of factors predicting treatment response, and the refinement of patient selection criteria to further enhance the therapeutic potential of this technique.

## Conclusion

The study conclusively demonstrates that laser ablation is a highly effective treatment for reducing the volume of thyroid nodules. With significant volume reductions observed at various post-procedure intervals, this minimally invasive technique shows promise for patients seeking alternatives to surgical intervention. The varying degrees of effectiveness based on initial nodule size, highlight the potential for tailored treatment approaches. Laser ablation thus offers a valuable addition to the therapeutic arsenal for thyroid nodules, combining efficacy with a favorable safety profile. This technique stands as a transformative approach in the management of thyroid nodules, aligning with the evolving landscape of patient-centered, minimally invasive medical care.

## Data Availability

The raw data supporting the conclusions of this article will be made available by the authors, without undue reservation.
