# Supplementary figures and images for: Efficacy of echolaser smart interface-guided laser ablation in volume reduction of symptomatic benign thyroid nodules
Source: Front Endocrinol (Lausanne). 2024 Oct 9;15:1402522. doi: 10.3389/fendo.2024.1402522 (PMC11496115; doi:10.3389/fendo.2024.1402522)

## Supplementary Material

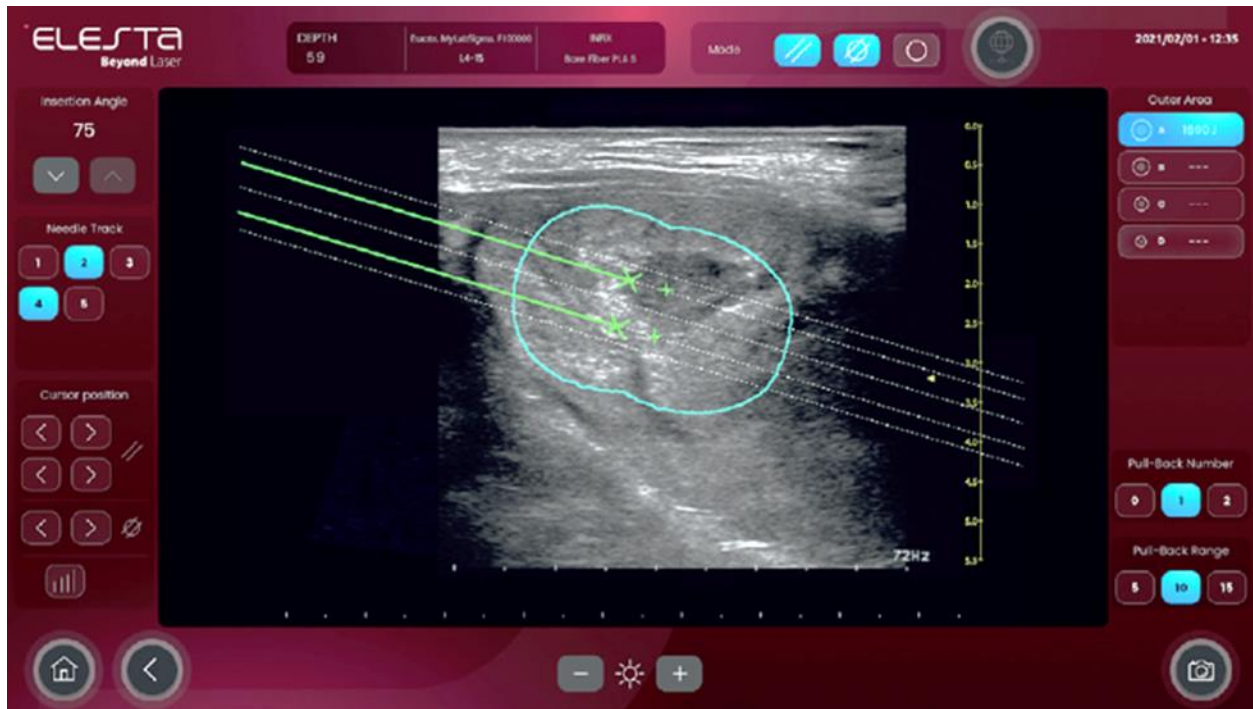

EchoLaser SmartInterface for planning laser ablation.

Supplement: Supplementary file 1 [file DataSheet1.pdf]
